# Supplementary figures and images for: Possible Reaction Mechanisms Involved in Degradation of Patulin by Heat-Assisted Cysteine under Highly Acidic Conditions
Source: Toxins (Basel). 2022 Oct 10;14(10):695. doi: 10.3390/toxins14100695 (PMC9610101; doi:10.3390/toxins14100695)

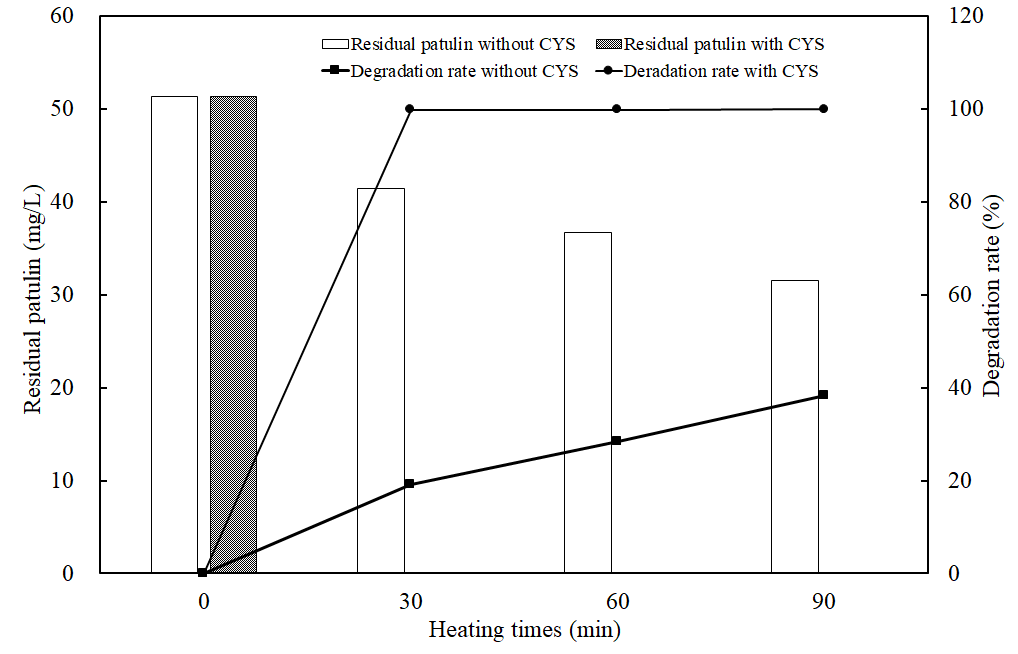

Supplement: Supplementary file 1 [file toxins-14-00695-s001.zip › Figure S1.tif]

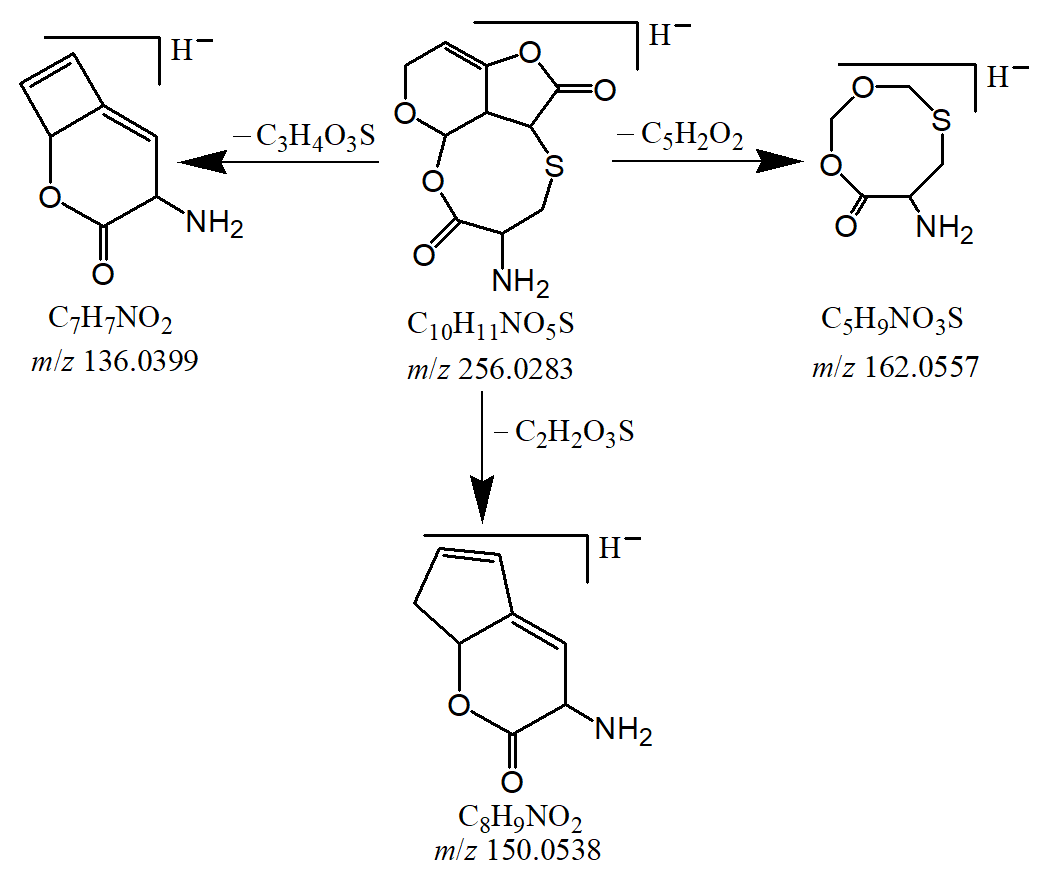

Supplement: Supplementary file 1 [file toxins-14-00695-s001.zip › Figure S2.tif]

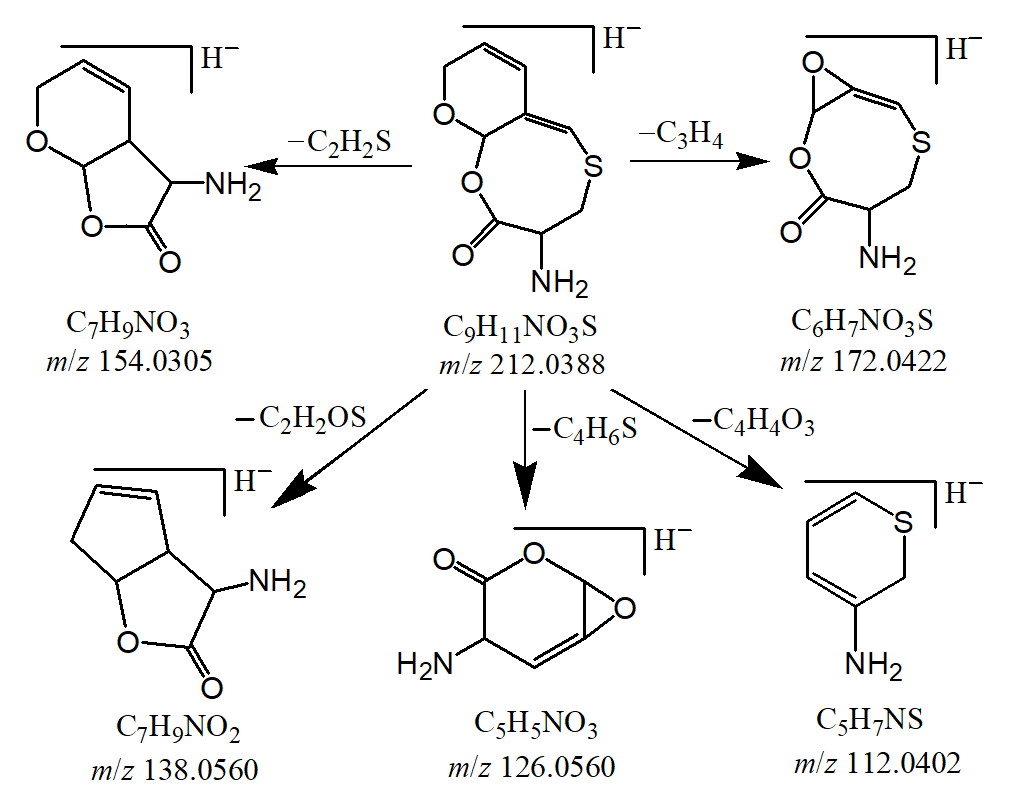

Supplement: Supplementary file 1 [file toxins-14-00695-s001.zip › Figure S3.tif]

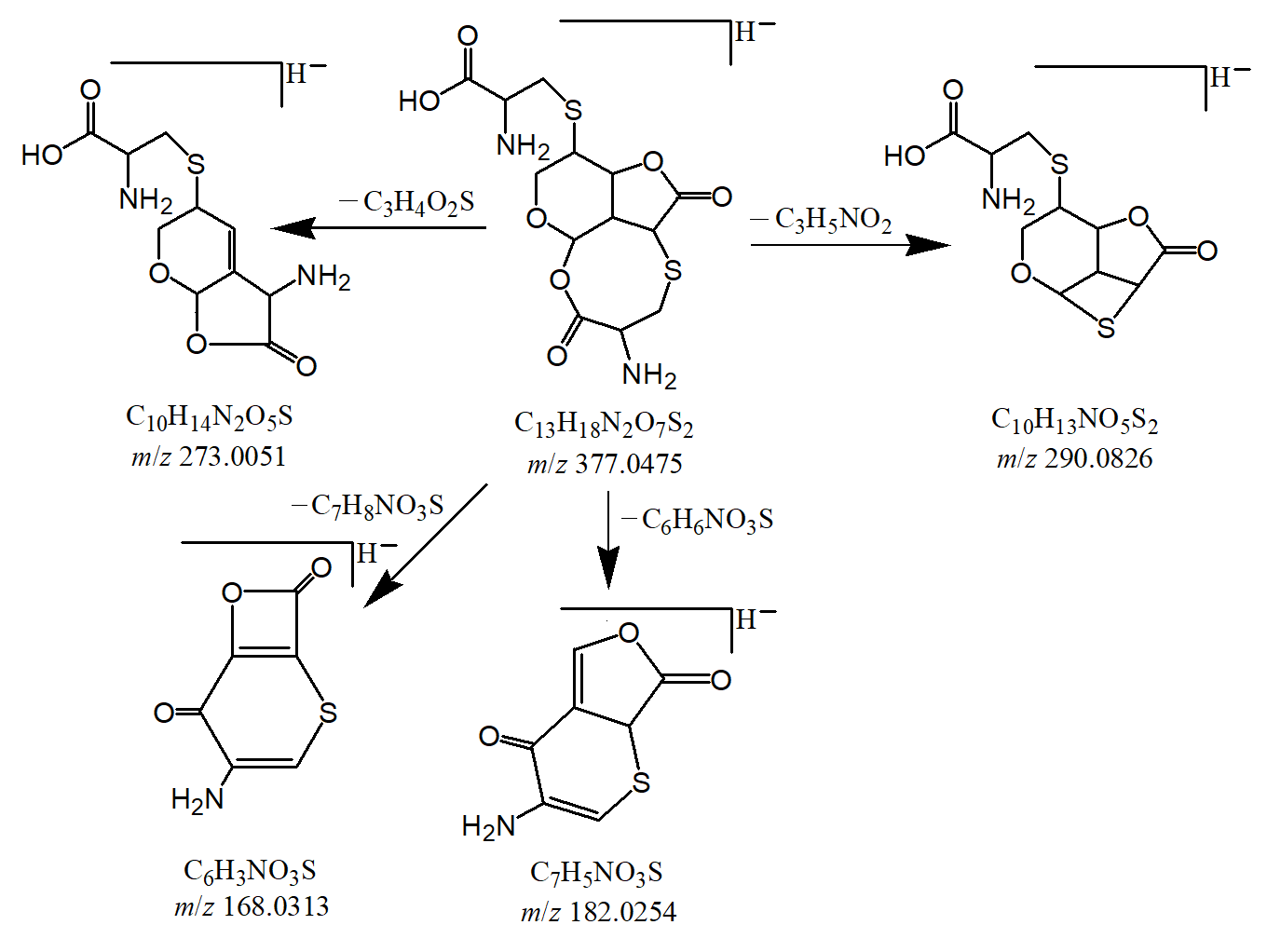

Supplement: Supplementary file 1 [file toxins-14-00695-s001.zip › Figure S4.tif]

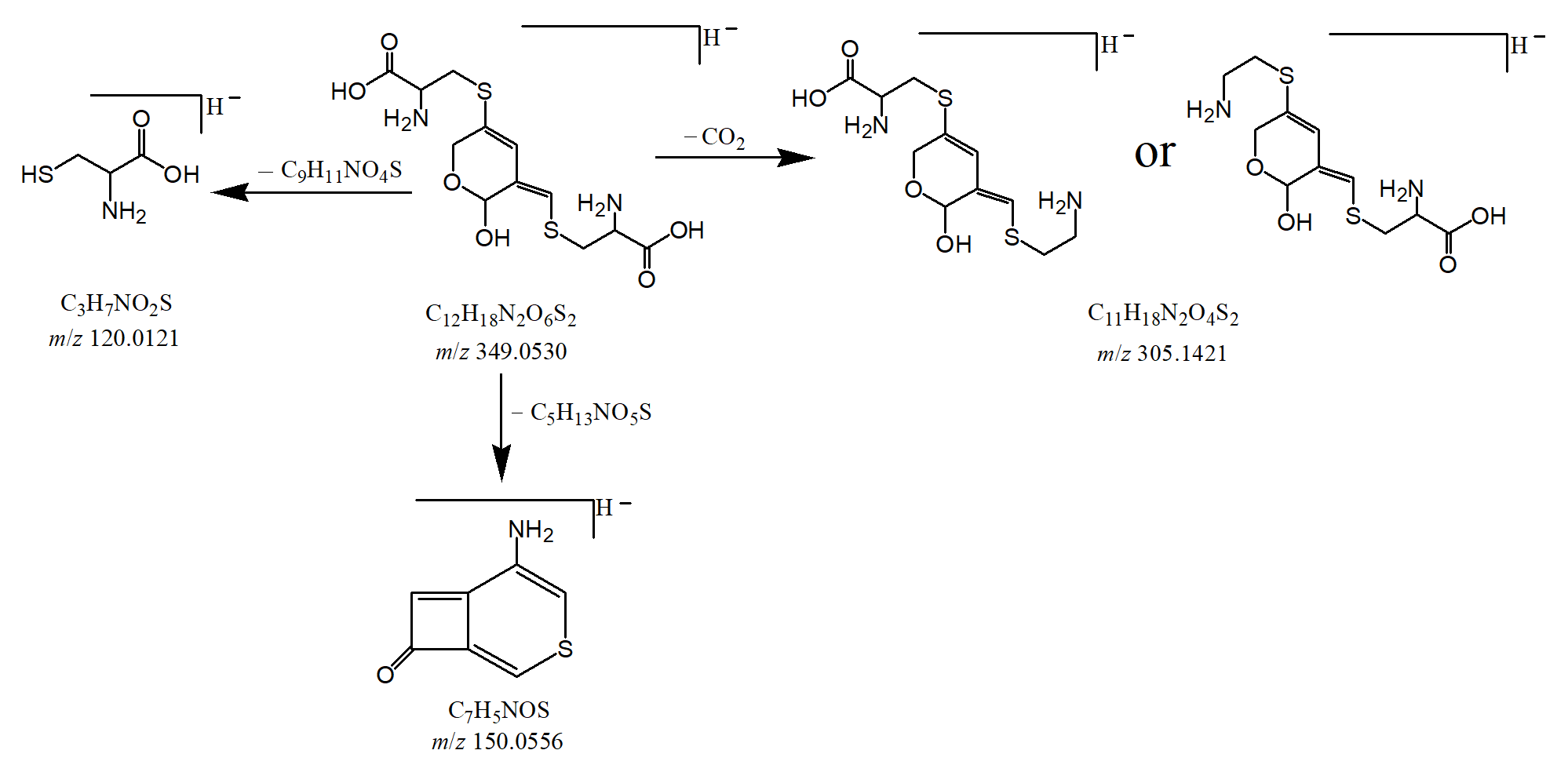

Supplement: Supplementary file 1 [file toxins-14-00695-s001.zip › Figure S5.tif]

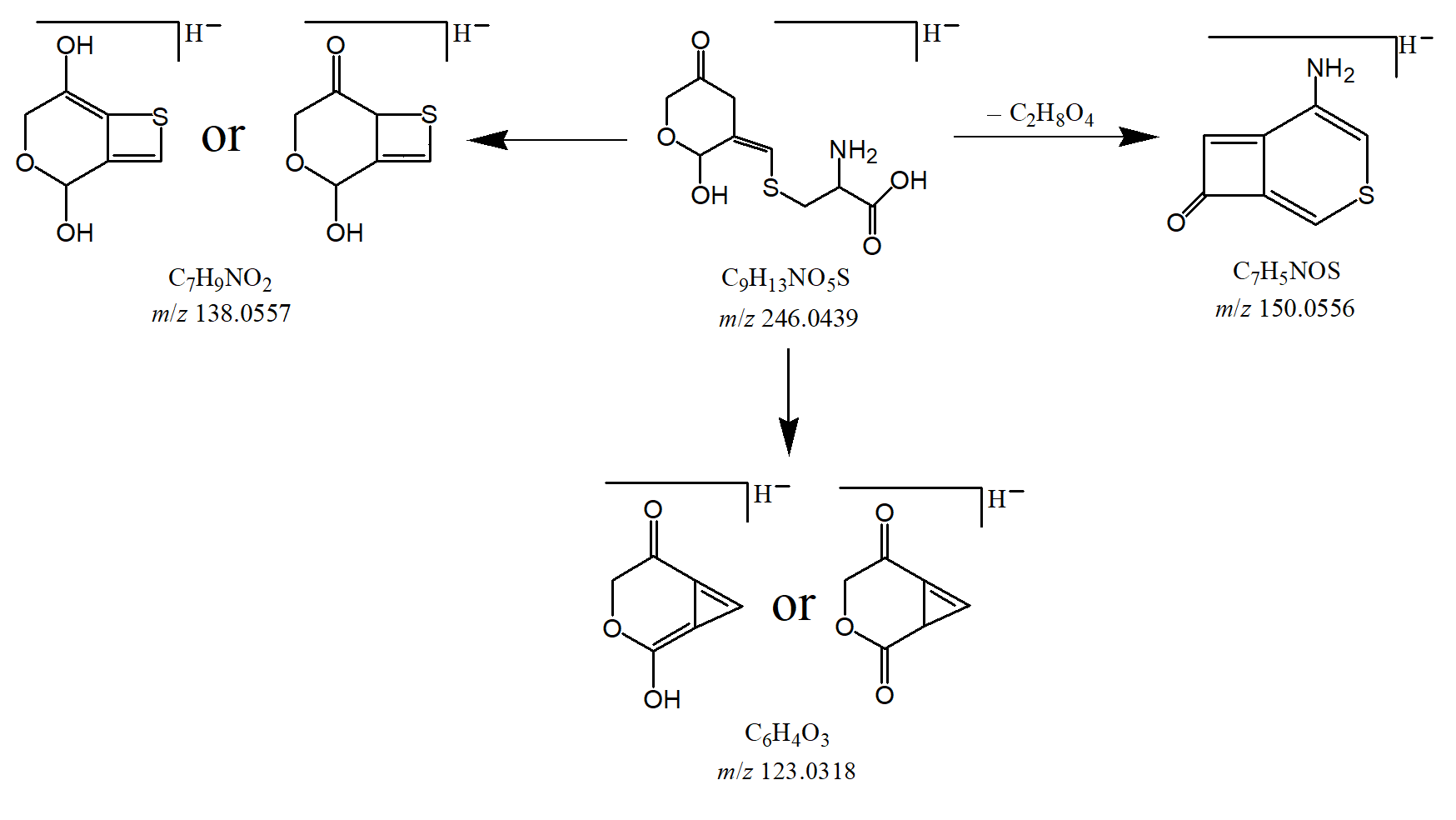

Supplement: Supplementary file 1 [file toxins-14-00695-s001.zip › Figure S6.tif]

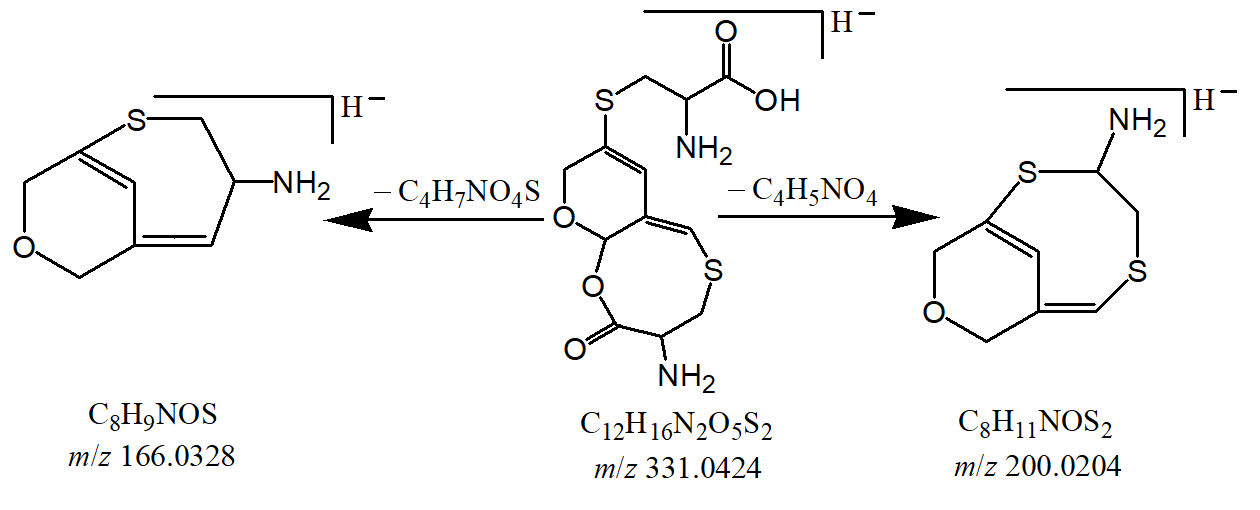

Supplement: Supplementary file 1 [file toxins-14-00695-s001.zip › Figure S7.tif]

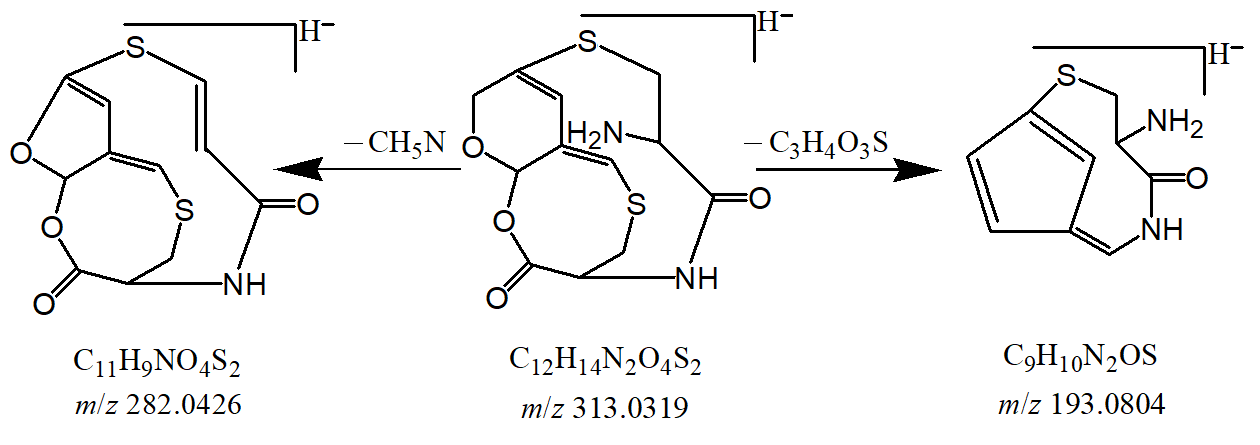

Supplement: Supplementary file 1 [file toxins-14-00695-s001.zip › Figure S8.tif]

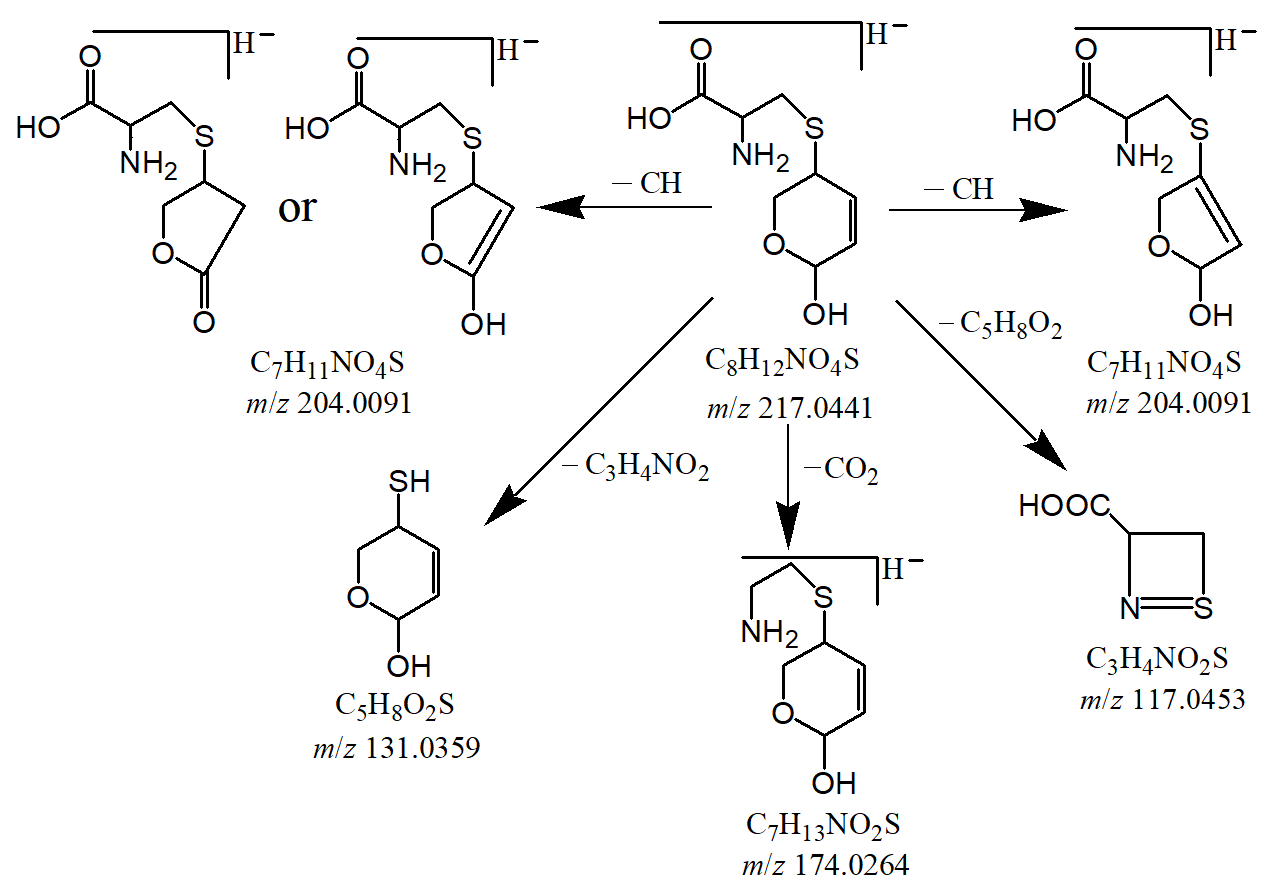

Supplement: Supplementary file 1 [file toxins-14-00695-s001.zip › Figure S9.tif]
